# (η^5^-Penta­methyl­cyclo­penta­dien­yl)(η^6^-4-phenyl­butan-2-one)ruthenium(II) tetra­phenyl­borate

**DOI:** 10.1107/S1600536810046064

**Published:** 2010-11-13

**Authors:** Bradley T. Loughrey, Michael L. Williams, Peter C. Healy

**Affiliations:** a Eskitis Institute for Cell and Molecular Therapies, Griffith University, Brisbane 4111, Australia; b School of Biomolecular and Physical Sciences, Griffith University, Brisbane 4111, Australia

## Abstract

The title compound, [Ru(C_10_H_15_)(C_10_H_12_O)][B(C_6_H_5_)_4_], crystallizes as discrete (η^5^-penta­methyl­cyclo­penta­dien­yl)Ru(η^6^-4-phenyl­butan-2-one)]^+^ cations and [BPh_4_]^−^ anions. In the cation, the non-H atoms of the butan-2-one group are approximately planar (r.m.s. deviation = 0.056 Å) and lie nearly perpendicular to the plane of the phenyl ring with a dihedral angle between the two planes of 69.3 (1)°. No significant C—H⋯O inter­actions are observed between the methyl and phenyl H atoms and the carbonyl O atom.

## Related literature

For related structures, see: Loughrey *et al.* (2008 ▶, 2009 ▶, 2010 ▶).
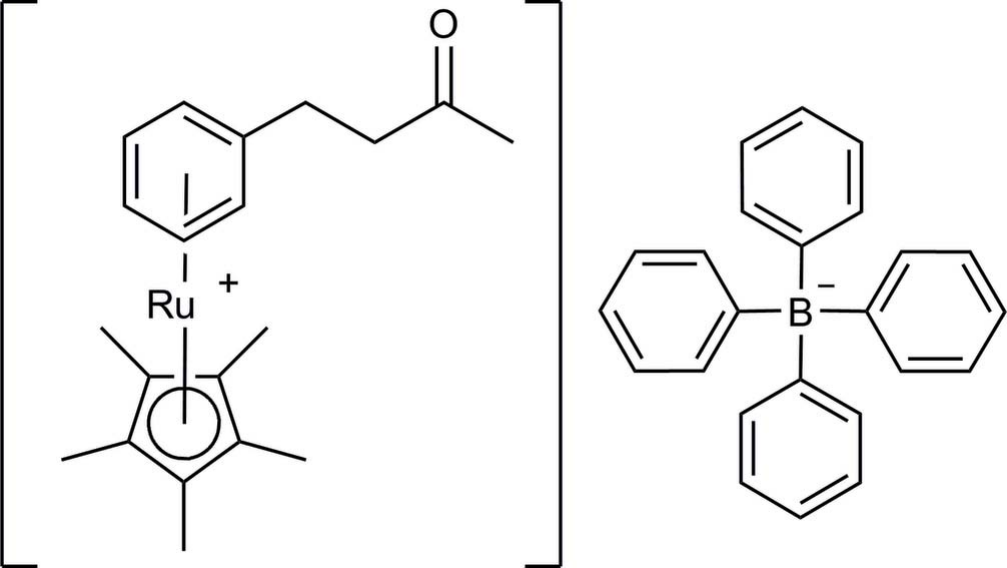


## Experimental

### 

#### Crystal data

[Ru(C_10_H_15_)(C_10_H_12_O)](C_24_H_20_B)
*M* *_r_* = 703.70
Monoclinic, 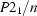

*a* = 13.1659 (2) Å
*b* = 19.7064 (2) Å
*c* = 14.3720 (2) Å
β = 92.378 (1)°
*V* = 3725.64 (9) Å^3^
*Z* = 4
Mo *K*α radiation
μ = 0.45 mm^−1^
*T* = 200 K
0.41 × 0.32 × 0.22 mm

#### Data collection

Oxford Diffraction Gemini S Ultra diffractometer
Absorption correction: multi-scan (*CrysAlis PRO*; Oxford Diffraction, 2010 ▶) *T*_min_ = 0.836, *T*_max_ = 0.907
20468 measured reflections
8549 independent reflections
7103 reflections with *I* > 2σ(*I*)
*R*_int_ = 0.016

#### Refinement

*R*[*F* ^2^ > 2σ(*F* ^2^)] = 0.025
*wR*(*F* ^2^) = 0.069
*S* = 1.10
8549 reflections
424 parameters
H-atom parameters constrained
Δρ_max_ = 0.54 e Å^−3^
Δρ_min_ = −0.55 e Å^−3^

### 

Data collection: *CrysAlis PRO* (Oxford Diffraction, 2010 ▶); cell refinement: *CrysAlis PRO*; data reduction: *CrysAlis PRO*; program(s) used to solve structure: *SIR97* (Altomare *et al.*, 1999 ▶); program(s) used to refine structure: *SHELXL97* (Sheldrick, 2008 ▶); molecular graphics: *ORTEP-3 for Windows* (Farrugia, 1997 ▶); software used to prepare material for publication: *PLATON* (Spek, 2009 ▶) and *publCIF* (Westrip, 2010 ▶).

## Supplementary Material

Crystal structure: contains datablocks global, I. DOI: 10.1107/S1600536810046064/bv2166sup1.cif

Structure factors: contains datablocks I. DOI: 10.1107/S1600536810046064/bv2166Isup2.hkl

Additional supplementary materials:  crystallographic information; 3D view; checkCIF report

## Figures and Tables

**Fig. 1.**
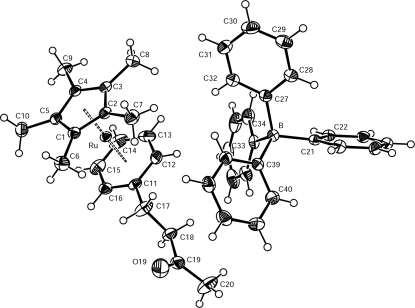
The structure of the cation and anion of (I), with atom labels and 40% probability displacement ellipsoids for the non-H atoms.

## supplementary crystallographic information

### Comment

As part of our ongoing investigations into the structural chemistry of ionic Ru(II) organometallic complex salts [Cp*Ru(II)-arene]^+^*X*^-^ (Loughrey *et al.*, 2008, 2009, 2010), we have determined the crystal structure of the complex of Cp*Ru and benzylacetone (PhCH_2_CH_2_C(=O)CH_3_) as the tetraphenylborate salt, (I) (Fig.1). Crystal packing in the structure is determined primarily by van der Waals and /C—H···π(Ph) interactions between the methyl and phenyl groups of the cation and the anion. No significant inter-molecular C—H···O interactions are observed between the carbonyl oxygen and methyl or phenyl H atoms.

The Cp* and benzene rings of the cation lie parallel (dihedral angle 1.5 (1) Å). The Ru—C(arene) bond lengths range between 2.207 (2) and 2.231 (2)Å with an average value of 2.216 (7) Å; and are comparable to those reported for related complexes (Loughrey *et al.*, 2010, and references therein).

The cation of (I) is isomeric with the propiophenone (PhC(=O)—Pr) complex, (Loughrey *et al.*, 2008). In this latter structure, the –C(=O)Pr group is co-planar with benzene ring with the carbonyl oxygen involved in an intra-molecular C—H···O interaction (O···H = 2.51 Å) with one *ortho* H atom on the benzene ring. In (I), the EtC(=O)Me group is also planar (r.m.s. deviation 0.056 Å) but steric interaction between the methylene protons and benzene protons and Cp* methyl protons result in the –C(=O)Pr group being oriented approximately orthogonal to the benzene ring with a dihedral angle of 110.7 (1)° between the two planes.

### Experimental

Benzylacetone (0.5 ml, 3.41 mmol) and HCp* (0.3 ml, 1.88 mmol) was added to a solution of ruthenium trichloride hydrate (0.20 g, 0.76 mmol) in ethanol (20 ml) under argon. The resulting solution was heated under reflux conditions for a period of 12 h to yield a golden-brown coloured solution. The solvent was concentrated *in vacuo* with the remaining residue being redissolved in acetone (20 ml) and merged with an acetone solution of sodium tetraphenylborate (5 ml, 0.30 *M*). The resulting mixture was concentrated to a minimum volume *in vacuo* and titrated with cold water to prompt precipitation of a tan powder. This powder was filtered from solution and washed with three aliquots of diethyl ether (20 ml) prior to being collected, and redissolved in a minimum quantity of acetone. This solution was filtered through a short alumina column (neutral, 150 mesh) using acetone as the eluent. The solution was concentrated *in vacuo* and the product recrystallized through addition of a minimum quantity of cold water. The resulting white, crystalline precipitate was then filtered from solution and dried *in vacuo*. Yield = 0.273 g, 72.3%. Large crystals suitable for X-ray diffraction studies were grown by slow diffusion of diethyl ether into a solution of the compound in acetone.

NMR: ^1^H (d_6_DMSO), δ 1.90 (s, 15H, C_5_(C_5_H_15_)), 2.08 (s, 3H, CH_3_), 2.47 (t, *J* = 8.0 Hz, 2H, C_6_H_5_—C*H*_2_), 2.76 (t, *J* = 8.0 Hz, 2H, CH_2_—CO), 5.79–5.86 (m, 5H, C_6_H_5_), 6.76–6.80 (m, 4H, B(C_6_H_5_)_4_*para*), 6.90–6.93 (m, 8H, B(C_6_H_5_)_4_*meta*), 7.16–7.19 (m, 8H, B(C_6_H_5_)_4_*ortho*); ^13^C (d_6_DMSO), δ 10.02 (C_5_(C_5_H_15_)), 26.08 (CH_3_),29.67 (C_6_H_5_-*C*H_2_), 43.47 (*C*H_2_—CO), 86.50 (1CH, aromatic), 87.09 (2CH, aromatic), 87.59 (2CH, aromatic), 95.35 (*C*_5_(C_5_H_15_)), 102.90 (1CH, aromatic), 121.47 (4CH, B(C_6_H_5_)_4_), 125.24 (8CH, B(C_6_H_5_)_4_), 135.49 (8CH, B(C_6_H_5_)_4_), 162.59, 163.08, 163.57, 164.06 (4CH, B(C_6_H_5_)_4_), signals split by ^11^B), 206.81(C=O); ESMS (m/*z*): +ve ion, calcd m/*z* for [(η^5^-C_5_(CH_3_)_5_)Ru(η^6^—C_6_H_5_(CH_2_)_2_COCH_3_)^+^]: 384.54, found: 385.09 (100%), -ve ion, calcd m/*z* for B(C_6_H_5_)_4_^-^: 319.25, found: 319.09 (100%); calcd % for C_44_H_47_OBRu: C 75.1, H 6.75%; found: C 75.0, H 6.79%.

### Refinement

All methyl hydrogen atoms were located from difference Fourier Maps. H atoms attached to carbon were constrained as riding atoms, with C–H set to 0.94–96 Å. *U*_iso_(H) values were set to 1.2*U*_eq_ (aromatic) and 1.5*U*_eq_ (alkyl) of the parent atom.

### Crystal data

**Table:**

[Ru(C_10_H_15_)(C_10_H_12_O)](C_24_H_20_B)	*F*(000) = 1472
*M**_r_* = 703.70	*D*_x_ = 1.255 Mg m^−^^3^
Monoclinic, *P*2_1_/*n*	Mo *K*α radiation, λ = 0.71070 Å
Hall symbol: -P 2yn	Cell parameters from 15293 reflections
*a* = 13.1659 (2) Å	θ = 3.1–32.2°
*b* = 19.7064 (2) Å	µ = 0.45 mm^−^^1^
*c* = 14.3720 (2) Å	*T* = 200 K
β = 92.378 (1)°	Block, colourless
*V* = 3725.64 (9) Å^3^	0.41 × 0.32 × 0.22 mm
*Z* = 4	

### Data collection

**Table:**

Oxford Diffraction Gemini S Ultra diffractometer	8549 independent reflections
Radiation source: Enhance (Mo) X-ray Source	7103 reflections with *I* > 2σ(*I*)
graphite	*R*_int_ = 0.016
Detector resolution: 16.0774 pixels mm^-1^	θ_max_ = 27.5°, θ_min_ = 3.1°
ω and φ scans	*h* = −15→17
Absorption correction: multi-scan (*CrysAlis PRO*; Oxford Diffraction, 2010)	*k* = −25→12
*T*_min_ = 0.836, *T*_max_ = 0.907	*l* = −18→8
20468 measured reflections	

### Refinement

**Table:**

Refinement on *F*^2^	Primary atom site location: structure-invariant direct methods
Least-squares matrix: full	Secondary atom site location: difference Fourier map
*R*[*F*^2^ > 2σ(*F*^2^)] = 0.025	Hydrogen site location: inferred from neighbouring sites
*wR*(*F*^2^) = 0.069	H-atom parameters constrained
*S* = 1.10	*w* = 1/[σ^2^(*F*_o_^2^) + (0.0396*P*)^2^ + 0.1643*P*] where *P* = (*F*_o_^2^ + 2*F*_c_^2^)/3
8549 reflections	(Δ/σ)_max_ = 0.001
424 parameters	Δρ_max_ = 0.54 e Å^−^^3^
0 restraints	Δρ_min_ = −0.55 e Å^−^^3^

### Special details

**Table:**

Experimental. CrysAlisPro (2010). Version 1.171.33.55 Oxford Diffraction Ltd., Empirical absorption correction using spherical harmonics, implemented in SCALE3 ABSPACK scaling algorithm.
Geometry. Bond distances, angles *etc*. have been calculated using the rounded fractional coordinates. All su's are estimated from the variances of the (full) variance-covariance matrix. The cell e.s.d.'s are taken into account in the estimation of distances, angles and torsion angles
Refinement. Refinement on *F*^2^ for ALL reflections except those flagged by the user for potential systematic errors. Weighted *R*-factors *wR* and all goodnesses of fit *S* are based on *F*^2^, conventional *R*-factors *R* are based on *F*, with *F* set to zero for negative *F*^2^. The observed criterion of *F*^2^ > σ(*F*^2^) is used only for calculating -*R*-factor-obs *etc*. and is not relevant to the choice of reflections for refinement. *R*-factors based on *F*^2^ are statistically about twice as large as those based on *F*, and *R*-factors based on ALL data will be even larger.

### Fractional atomic coordinates and isotropic or equivalent isotropic displacement parameters (Å^2^)

**Table:**

	*x*	*y*	*z*	*U*_iso_*/*U*_eq_	
Ru	0.55316 (1)	0.14771 (1)	0.23154 (1)	0.0247 (1)	
O19	0.19486 (12)	−0.00976 (8)	0.05380 (11)	0.0631 (6)	
C1	0.61274 (13)	0.13342 (8)	0.09337 (11)	0.0282 (4)	
C2	0.56126 (12)	0.19729 (8)	0.09612 (11)	0.0284 (5)	
C3	0.61217 (12)	0.23839 (8)	0.16627 (11)	0.0300 (5)	
C4	0.69582 (12)	0.20024 (8)	0.20580 (11)	0.0291 (5)	
C5	0.69633 (12)	0.13513 (8)	0.16034 (11)	0.0287 (5)	
C6	0.58693 (15)	0.07559 (9)	0.02864 (11)	0.0387 (5)	
C7	0.47463 (14)	0.21912 (9)	0.03281 (12)	0.0405 (6)	
C8	0.58342 (15)	0.30941 (9)	0.19115 (14)	0.0435 (6)	
C9	0.77350 (14)	0.22424 (9)	0.27776 (13)	0.0419 (6)	
C10	0.77516 (14)	0.08114 (9)	0.17554 (13)	0.0421 (6)	
C11	0.40664 (12)	0.09177 (8)	0.24079 (11)	0.0302 (5)	
C12	0.39532 (15)	0.15832 (9)	0.27675 (14)	0.0414 (6)	
C13	0.46368 (18)	0.18242 (10)	0.34902 (14)	0.0524 (7)	
C14	0.54139 (17)	0.14065 (12)	0.38474 (12)	0.0519 (7)	
C15	0.55284 (14)	0.07558 (11)	0.34985 (12)	0.0445 (6)	
C16	0.48612 (14)	0.05140 (8)	0.27914 (11)	0.0341 (5)	
C17	0.33680 (15)	0.06448 (12)	0.16405 (12)	0.0488 (6)	
C18	0.23631 (14)	0.04201 (9)	0.20012 (12)	0.0391 (5)	
C19	0.16669 (14)	0.00779 (9)	0.12875 (14)	0.0416 (6)	
C20	0.05998 (18)	−0.00489 (15)	0.15720 (19)	0.0739 (10)	
C21	−0.00388 (12)	0.34793 (7)	0.19919 (10)	0.0250 (4)	
C22	−0.02878 (13)	0.36758 (8)	0.28945 (11)	0.0289 (4)	
C23	−0.12081 (13)	0.39848 (8)	0.30875 (11)	0.0332 (5)	
C24	−0.19288 (13)	0.41071 (8)	0.23771 (13)	0.0354 (5)	
C25	−0.17137 (13)	0.39159 (8)	0.14789 (12)	0.0350 (5)	
C26	−0.07942 (13)	0.36100 (8)	0.12939 (11)	0.0311 (5)	
C27	0.18052 (12)	0.37759 (8)	0.13734 (10)	0.0278 (4)	
C28	0.14028 (13)	0.43584 (8)	0.09393 (10)	0.0311 (5)	
C29	0.20017 (16)	0.48550 (9)	0.05478 (12)	0.0405 (6)	
C30	0.30419 (17)	0.47859 (10)	0.05630 (14)	0.0486 (7)	
C31	0.34797 (15)	0.42240 (10)	0.09943 (15)	0.0474 (6)	
C32	0.28726 (14)	0.37357 (9)	0.13994 (13)	0.0378 (5)	
C33	0.15746 (12)	0.28317 (8)	0.27402 (10)	0.0295 (4)	
C34	0.21760 (14)	0.32051 (10)	0.33815 (12)	0.0406 (6)	
C35	0.25258 (15)	0.29418 (13)	0.42355 (13)	0.0527 (7)	
C36	0.22896 (17)	0.22937 (13)	0.44790 (13)	0.0569 (8)	
C37	0.16987 (18)	0.19048 (11)	0.38736 (14)	0.0538 (7)	
C38	0.13437 (15)	0.21704 (9)	0.30198 (12)	0.0389 (6)	
C39	0.10006 (12)	0.25499 (7)	0.09834 (10)	0.0263 (4)	
C40	0.01493 (14)	0.21305 (9)	0.08473 (12)	0.0381 (5)	
C41	0.01377 (16)	0.15765 (9)	0.02438 (15)	0.0466 (6)	
C42	0.09801 (16)	0.14178 (9)	−0.02492 (13)	0.0440 (6)	
C43	0.18323 (14)	0.18193 (9)	−0.01415 (12)	0.0391 (6)	
C44	0.18349 (13)	0.23738 (8)	0.04559 (11)	0.0333 (5)	
B	0.10817 (14)	0.31626 (9)	0.17692 (11)	0.0257 (5)	
H6A	0.60050	0.03380	0.05970	0.0460*	
H6B	0.62700	0.07860	−0.02470	0.0460*	
H6C	0.51690	0.07770	0.00990	0.0460*	
H7A	0.43160	0.18140	0.01930	0.0490*	
H7B	0.50010	0.23620	−0.02340	0.0490*	
H7C	0.43710	0.25360	0.06220	0.0490*	
H8A	0.61310	0.32060	0.25050	0.0520*	
H8B	0.51150	0.31280	0.19280	0.0520*	
H8C	0.60740	0.33990	0.14580	0.0520*	
H9A	0.80030	0.18640	0.31160	0.0500*	
H9B	0.74250	0.25460	0.31930	0.0500*	
H9C	0.82690	0.24680	0.24790	0.0500*	
H10A	0.80160	0.08310	0.23800	0.0510*	
H10B	0.74530	0.03790	0.16410	0.0510*	
H10C	0.82860	0.08810	0.13420	0.0510*	
H12	0.34220	0.18680	0.25280	0.0500*	
H13	0.45630	0.22700	0.37280	0.0630*	
H14	0.58660	0.15670	0.43310	0.0620*	
H15	0.60610	0.04740	0.37400	0.0530*	
H16	0.49470	0.00660	0.25640	0.0410*	
H17A	0.36850	0.02680	0.13600	0.0580*	
H17B	0.32490	0.09900	0.11880	0.0580*	
H18A	0.20240	0.08090	0.22270	0.0470*	
H18B	0.24950	0.01110	0.24990	0.0470*	
H20A	0.01500	−0.00300	0.10380	0.0890*	
H20B	0.04130	0.02880	0.20060	0.0890*	
H20C	0.05610	−0.04850	0.18510	0.0890*	
H22	0.01920	0.35950	0.33940	0.0350*	
H23	−0.13420	0.41120	0.37080	0.0400*	
H24	−0.25570	0.43180	0.25030	0.0420*	
H25	−0.22010	0.39950	0.09850	0.0420*	
H26	−0.06700	0.34840	0.06710	0.0370*	
H28	0.06860	0.44170	0.09110	0.0370*	
H29	0.16910	0.52440	0.02680	0.0490*	
H30	0.34530	0.51190	0.02830	0.0580*	
H31	0.41970	0.41710	0.10140	0.0570*	
H32	0.31930	0.33600	0.17050	0.0450*	
H34	0.23530	0.36580	0.32280	0.0490*	
H35	0.29310	0.32150	0.46500	0.0630*	
H36	0.25300	0.21130	0.50600	0.0680*	
H37	0.15310	0.14530	0.40370	0.0650*	
H38	0.09330	0.18940	0.26150	0.0470*	
H40	−0.04430	0.22270	0.11780	0.0460*	
H41	−0.04570	0.13060	0.01730	0.0560*	
H42	0.09730	0.10380	−0.06570	0.0530*	
H43	0.24200	0.17170	−0.04760	0.0470*	
H44	0.24290	0.26470	0.05100	0.0400*	

### Atomic displacement parameters (Å^2^)

**Table:**

	*U*^11^	*U*^22^	*U*^33^	*U*^12^	*U*^13^	*U*^23^
Ru	0.0247 (1)	0.0262 (1)	0.0235 (1)	−0.0051 (1)	0.0057 (1)	−0.0032 (1)
O19	0.0548 (10)	0.0756 (10)	0.0578 (9)	0.0044 (8)	−0.0102 (7)	−0.0266 (8)
C1	0.0303 (8)	0.0298 (8)	0.0252 (7)	−0.0038 (6)	0.0091 (6)	0.0007 (6)
C2	0.0248 (8)	0.0318 (8)	0.0291 (8)	−0.0038 (6)	0.0062 (6)	0.0032 (6)
C3	0.0251 (8)	0.0293 (8)	0.0360 (8)	−0.0046 (6)	0.0069 (6)	0.0020 (6)
C4	0.0253 (8)	0.0308 (8)	0.0316 (8)	−0.0052 (6)	0.0060 (6)	0.0013 (6)
C5	0.0279 (8)	0.0294 (8)	0.0294 (8)	−0.0011 (6)	0.0091 (6)	0.0038 (6)
C6	0.0484 (11)	0.0393 (9)	0.0288 (8)	−0.0027 (8)	0.0069 (7)	−0.0060 (7)
C7	0.0355 (10)	0.0458 (10)	0.0400 (9)	−0.0005 (8)	−0.0017 (7)	0.0063 (8)
C8	0.0383 (10)	0.0293 (9)	0.0630 (12)	−0.0031 (8)	0.0052 (9)	−0.0037 (8)
C9	0.0328 (10)	0.0456 (10)	0.0469 (10)	−0.0116 (8)	−0.0025 (8)	−0.0019 (8)
C10	0.0364 (10)	0.0413 (10)	0.0491 (10)	0.0063 (8)	0.0081 (8)	0.0057 (8)
C11	0.0275 (8)	0.0370 (9)	0.0267 (7)	−0.0097 (7)	0.0074 (6)	−0.0004 (6)
C12	0.0343 (10)	0.0371 (10)	0.0546 (11)	0.0014 (8)	0.0227 (8)	0.0030 (8)
C13	0.0680 (14)	0.0399 (10)	0.0525 (12)	−0.0231 (10)	0.0395 (11)	−0.0219 (9)
C14	0.0508 (12)	0.0801 (15)	0.0254 (9)	−0.0281 (11)	0.0093 (8)	−0.0056 (9)
C15	0.0383 (10)	0.0628 (12)	0.0324 (9)	−0.0071 (9)	0.0021 (7)	0.0164 (9)
C16	0.0386 (9)	0.0308 (8)	0.0338 (8)	−0.0064 (7)	0.0112 (7)	0.0034 (7)
C17	0.0375 (10)	0.0758 (14)	0.0329 (9)	−0.0224 (10)	0.0001 (7)	0.0000 (9)
C18	0.0318 (9)	0.0435 (10)	0.0419 (9)	−0.0103 (8)	0.0013 (7)	−0.0047 (8)
C19	0.0363 (10)	0.0332 (9)	0.0544 (11)	−0.0016 (8)	−0.0089 (8)	−0.0020 (8)
C20	0.0420 (13)	0.0947 (19)	0.0840 (17)	−0.0256 (13)	−0.0093 (12)	−0.0062 (14)
C21	0.0275 (8)	0.0217 (7)	0.0260 (7)	−0.0024 (6)	0.0023 (6)	0.0022 (6)
C22	0.0318 (8)	0.0270 (8)	0.0279 (7)	−0.0028 (6)	0.0013 (6)	−0.0011 (6)
C23	0.0351 (9)	0.0289 (8)	0.0362 (9)	−0.0044 (7)	0.0088 (7)	−0.0065 (7)
C24	0.0284 (9)	0.0245 (8)	0.0537 (10)	−0.0006 (7)	0.0085 (7)	−0.0017 (7)
C25	0.0290 (9)	0.0340 (9)	0.0415 (9)	0.0004 (7)	−0.0030 (7)	0.0092 (7)
C26	0.0326 (9)	0.0341 (9)	0.0267 (8)	0.0005 (7)	0.0011 (6)	0.0035 (6)
C27	0.0327 (9)	0.0268 (7)	0.0241 (7)	−0.0007 (7)	0.0024 (6)	−0.0040 (6)
C28	0.0381 (9)	0.0295 (8)	0.0258 (7)	−0.0009 (7)	0.0011 (6)	−0.0016 (6)
C29	0.0566 (12)	0.0320 (9)	0.0332 (9)	−0.0041 (8)	0.0040 (8)	0.0040 (7)
C30	0.0596 (13)	0.0386 (10)	0.0487 (11)	−0.0145 (9)	0.0165 (9)	0.0030 (8)
C31	0.0360 (10)	0.0443 (11)	0.0626 (12)	−0.0070 (8)	0.0122 (9)	−0.0022 (9)
C32	0.0356 (10)	0.0311 (8)	0.0469 (10)	−0.0003 (7)	0.0049 (8)	0.0008 (7)
C33	0.0285 (8)	0.0331 (8)	0.0269 (7)	0.0088 (7)	0.0025 (6)	−0.0001 (6)
C34	0.0357 (10)	0.0521 (11)	0.0336 (9)	0.0059 (8)	−0.0045 (7)	−0.0026 (8)
C35	0.0388 (11)	0.0869 (16)	0.0317 (9)	0.0182 (11)	−0.0067 (8)	−0.0035 (10)
C36	0.0507 (12)	0.0918 (17)	0.0284 (9)	0.0390 (12)	0.0054 (8)	0.0139 (10)
C37	0.0634 (14)	0.0550 (12)	0.0445 (11)	0.0292 (11)	0.0206 (10)	0.0215 (9)
C38	0.0460 (11)	0.0374 (9)	0.0340 (9)	0.0116 (8)	0.0088 (7)	0.0057 (7)
C39	0.0302 (8)	0.0253 (7)	0.0231 (7)	0.0018 (6)	−0.0012 (6)	0.0021 (6)
C40	0.0355 (9)	0.0367 (9)	0.0424 (9)	−0.0021 (8)	0.0063 (7)	−0.0064 (7)
C41	0.0431 (11)	0.0381 (10)	0.0582 (12)	−0.0082 (8)	−0.0027 (9)	−0.0128 (8)
C42	0.0525 (12)	0.0370 (10)	0.0419 (10)	0.0038 (9)	−0.0036 (8)	−0.0134 (8)
C43	0.0400 (10)	0.0413 (10)	0.0363 (9)	0.0076 (8)	0.0050 (7)	−0.0070 (7)
C44	0.0318 (9)	0.0332 (8)	0.0349 (8)	0.0015 (7)	0.0012 (7)	−0.0035 (7)
B	0.0283 (9)	0.0261 (8)	0.0228 (8)	0.0019 (7)	0.0007 (6)	0.0002 (6)

### Geometric parameters (Å, °)

**Table:**

Ru—C1	2.1834 (16)	C18—H18A	0.9500
Ru—C2	2.1844 (16)	C18—H18B	0.9500
Ru—C3	2.1766 (16)	C20—H20A	0.9500
Ru—C4	2.1896 (16)	C20—H20B	0.9500
Ru—C5	2.1959 (16)	C20—H20C	0.9500
Ru—C11	2.2305 (16)	C21—C22	1.406 (2)
Ru—C12	2.213 (2)	C21—C26	1.407 (2)
Ru—C13	2.207 (2)	C21—B	1.645 (2)
Ru—C14	2.2180 (17)	C22—C23	1.394 (2)
Ru—C15	2.2163 (19)	C23—C24	1.386 (2)
Ru—C16	2.2133 (16)	C24—C25	1.385 (3)
O19—C19	1.204 (3)	C25—C26	1.388 (2)
C1—C2	1.431 (2)	C27—C28	1.400 (2)
C1—C5	1.432 (2)	C27—C32	1.407 (2)
C1—C6	1.501 (2)	C27—B	1.654 (2)
C2—C3	1.437 (2)	C28—C29	1.390 (2)
C2—C7	1.493 (2)	C29—C30	1.376 (3)
C3—C4	1.431 (2)	C30—C31	1.383 (3)
C3—C8	1.497 (2)	C31—C32	1.394 (3)
C4—C5	1.440 (2)	C33—C34	1.399 (2)
C4—C9	1.501 (2)	C33—C38	1.401 (2)
C5—C10	1.496 (2)	C33—B	1.649 (2)
C11—C12	1.420 (2)	C34—C35	1.393 (3)
C11—C16	1.409 (2)	C35—C36	1.364 (4)
C11—C17	1.506 (2)	C36—C37	1.376 (3)
C12—C13	1.427 (3)	C37—C38	1.397 (3)
C13—C14	1.394 (3)	C39—C40	1.400 (2)
C14—C15	1.387 (3)	C39—C44	1.404 (2)
C15—C16	1.399 (2)	C39—B	1.654 (2)
C17—C18	1.507 (3)	C40—C41	1.394 (3)
C18—C19	1.506 (3)	C41—C42	1.377 (3)
C19—C20	1.500 (3)	C42—C43	1.376 (3)
C6—H6A	0.9500	C43—C44	1.390 (2)
C6—H6B	0.9500	C22—H22	0.9500
C6—H6C	0.9500	C23—H23	0.9500
C7—H7A	0.9500	C24—H24	0.9500
C7—H7B	0.9500	C25—H25	0.9500
C7—H7C	0.9500	C26—H26	0.9500
C8—H8A	0.9500	C28—H28	0.9500
C8—H8B	0.9500	C29—H29	0.9500
C8—H8C	0.9500	C30—H30	0.9500
C9—H9A	0.9500	C31—H31	0.9500
C9—H9B	0.9500	C32—H32	0.9500
C9—H9C	0.9500	C34—H34	0.9500
C10—H10A	0.9500	C35—H35	0.9500
C10—H10B	0.9500	C36—H36	0.9500
C10—H10C	0.9500	C37—H37	0.9500
C12—H12	0.9500	C38—H38	0.9500
C13—H13	0.9500	C40—H40	0.9500
C14—H14	0.9500	C41—H41	0.9500
C15—H15	0.9500	C42—H42	0.9500
C16—H16	0.9500	C43—H43	0.9500
C17—H17A	0.9500	C44—H44	0.9500
C17—H17B	0.9500		
			
O19···C6^i^	3.409 (2)	C28···H18B^viii^	3.0100
O19···H17A	2.6300	C29···H18B^viii^	2.9000
O19···H17B	2.8700	C29···H15^vii^	2.9000
O19···H6B^i^	2.7600	C30···H18B^viii^	2.9700
C1···C3	2.319 (2)	C32···H44	2.5500
C1···C4	2.323 (2)	C32···H34	2.7500
C1···C7	2.605 (2)	C33···H32	2.8500
C1···C10	2.611 (2)	C33···H22	2.5700
C1···C16	3.590 (2)	C34···H32	2.8200
C1···C35^ii^	3.429 (3)	C34···H6B^ix^	3.0800
C2···C4	2.323 (2)	C34···H22	2.7200
C2···C5	2.319 (2)	C37···H12	3.0400
C2···C6	2.614 (2)	C37···H7B^ix^	2.9900
C2···C8	2.608 (2)	C38···H18A	3.0600
C2···C12	3.546 (3)	C38···H12	2.9200
C2···C36^ii^	3.449 (3)	C39···H26	2.8900
C3···C1	2.319 (2)	C39···H14^vii^	2.9400
C3···C5	2.320 (2)	C39···H38	2.6800
C3···C7	2.610 (2)	C40···H26	2.8800
C3···C9	2.622 (2)	C40···H38	2.7400
C3···C13	3.516 (3)	C41···H35^vii^	3.0200
C4···C1	2.323 (2)	C43···H17B	3.0800
C4···C2	2.323 (2)	C43···H9B^vii^	2.8400
C4···C8	2.615 (2)	C44···H14^vii^	2.9000
C4···C10	2.613 (2)	H6A···C10	2.9300
C4···C14	3.544 (3)	H6A···H10B	2.3800
C5···C2	2.319 (2)	H6B···C34^ii^	3.0800
C5···C3	2.320 (2)	H6B···O19^i^	2.7600
C5···C6	2.609 (2)	H6C···H7A	2.3400
C5···C9	2.612 (2)	H6C···C7	2.8600
C5···C15	3.577 (2)	H7A···C6	2.9200
C6···O19^i^	3.409 (2)	H7A···H6C	2.3400
C8···C24^iii^	3.598 (3)	H7B···C37^ii^	2.9900
C11···C15	2.452 (2)	H7C···H44	2.5600
C11···C14	2.837 (3)	H7C···C8	2.8400
C11···C13	2.465 (3)	H7C···H8B	2.3900
C12···C15	2.808 (3)	H8A···C9	2.8500
C12···C37	3.483 (3)	H8A···H9B	2.3300
C12···C2	3.546 (3)	H8B···C7	2.9700
C12···C16	2.422 (2)	H8B···H7C	2.3900
C12···C14	2.446 (3)	H8B···H32	2.5800
C12···C17	2.556 (3)	H8C···C25^iii^	3.0800
C13···C11	2.465 (3)	H9A···H10A	2.2900
C13···C15	2.410 (3)	H9A···C10	2.8600
C13···C36	3.576 (3)	H9B···C43^iv^	2.8400
C13···C16	2.791 (3)	H9B···H43^iv^	2.4000
C13···C3	3.516 (3)	H9B···H8A	2.3300
C14···C28^iv^	3.561 (2)	H9B···C8	2.9400
C14···C16	2.416 (3)	H9C···C21^iii^	3.0900
C14···C12	2.446 (3)	H9C···C22^iii^	3.0900
C14···C4	3.544 (3)	H10A···H9A	2.2900
C14···C11	2.837 (3)	H10A···C9	2.8700
C15···C13	2.410 (3)	H10B···C6	2.8900
C15···C5	3.577 (2)	H10B···H6A	2.3800
C15···C11	2.452 (2)	H10B···C24^v^	2.9700
C15···C12	2.808 (3)	H10B···H24^v^	2.4300
C16···C12	2.422 (2)	H10C···H41^iii^	2.5500
C16···C28^v^	3.396 (2)	H12···H17B	2.5900
C16···C1	3.590 (2)	H12···C37	3.0400
C16···C17	2.529 (3)	H12···C38	2.9200
C16···C13	2.791 (3)	H14···C39^iv^	2.9400
C16···C14	2.416 (3)	H14···C44^iv^	2.9000
C19···C42	3.536 (3)	H14···C28^iv^	3.0100
C22···C34	3.418 (3)	H15···C29^iv^	2.9000
C24···C8^vi^	3.598 (3)	H16···C23^v^	2.8800
C26···C28	3.305 (2)	H16···H17A	2.3800
C26···C40	3.244 (2)	H16···C22^v^	2.8600
C28···C14^vii^	3.561 (2)	H17A···O19	2.6300
C28···C16^viii^	3.396 (2)	H17A···H16	2.3800
C28···C26	3.305 (2)	H17B···O19	2.8700
C32···C34	3.204 (3)	H17B···C43	3.0800
C32···C44	3.279 (2)	H17B···H12	2.5900
C34···C22	3.418 (3)	H18A···H20B	2.3700
C34···C32	3.204 (3)	H18A···C12	3.0400
C35···C1^ix^	3.429 (3)	H18A···C38	3.0600
C36···C2^ix^	3.449 (3)	H18B···C28^v^	3.0100
C36···C13	3.576 (3)	H18B···C30^v^	2.9700
C37···C12	3.483 (3)	H18B···C29^v^	2.9000
C38···C40	3.438 (2)	H20A···H42^x^	2.5200
C40···C26	3.244 (2)	H20B···H18A	2.3700
C40···C38	3.438 (2)	H22···C33	2.5700
C42···C19	3.536 (3)	H22···C34	2.7200
C44···C32	3.279 (2)	H24···H10B^viii^	2.4300
C6···H10B	2.8900	H25···H29^xi^	2.4600
C6···H7A	2.9200	H25···H36^vii^	2.5700
C7···H6C	2.8600	H26···C39	2.8900
C7···H8B	2.9700	H26···C40	2.8800
C8···H7C	2.8400	H26···H28	2.5800
C8···H9B	2.9400	H26···H40	2.6000
C9···H10A	2.8700	H26···C14^vii^	3.0400
C9···H8A	2.8500	H28···C21	2.6200
C10···H6A	2.9300	H28···C26	2.5900
C10···H9A	2.8600	H28···H26	2.5800
C11···H12	2.0700	H28···C16^viii^	2.9600
C11···H16	2.0500	H29···C25^xi^	3.0100
C12···H18A	3.0400	H29···H25^xi^	2.4600
C12···H13	2.0700	H32···C33	2.8500
C13···H12	2.0700	H32···C34	2.8200
C13···H14	2.0400	H32···H8B	2.5800
C14···H26^iv^	3.0400	H32···H34	2.5600
C14···H13	2.0400	H32···H44	2.4100
C14···H15	2.0300	H34···C27	2.7400
C15···H14	2.0300	H34···C32	2.7500
C15···H16	2.0400	H34···H32	2.5600
C16···H15	2.0400	H35···C41^iv^	3.0200
C16···H28^v^	2.9600	H35···H41^iv^	2.4100
C20···H42^x^	3.1000	H36···C25^iv^	3.0200
C21···H40	2.7700	H36···H25^iv^	2.5700
C21···H28	2.6200	H38···C39	2.6800
C21···H9C^vi^	3.0900	H38···C40	2.7400
C22···H16^viii^	2.8600	H40···C21	2.7700
C22···H9C^vi^	3.0900	H40···C26	2.7700
C23···H16^viii^	2.8800	H40···H26	2.6000
C24···H10B^viii^	2.9700	H41···H10C^vi^	2.5500
C25···H36^vii^	3.0200	H41···H35^vii^	2.4100
C25···H29^xi^	3.0100	H42···C20^x^	3.1000
C25···H8C^vi^	3.0800	H42···H20A^x^	2.5200
C26···H28	2.5900	H43···H9B^vii^	2.4000
C26···H40	2.7700	H44···C27	2.6900
C27···H34	2.7400	H44···C32	2.5500
C27···H44	2.6900	H44···H7C	2.5600
C28···H14^vii^	3.0100	H44···H32	2.4100
			
C1—Ru—C2	38.25 (6)	C3—C8—H8C	109.00
C1—Ru—C3	64.26 (6)	H8A—C8—H8B	110.00
C1—Ru—C4	64.16 (6)	H8A—C8—H8C	109.00
C1—Ru—C5	38.17 (6)	H8B—C8—H8C	109.00
C1—Ru—C11	109.56 (6)	C4—C9—H9A	109.00
C1—Ru—C12	131.03 (7)	C4—C9—H9B	109.00
C1—Ru—C13	163.54 (7)	C4—C9—H9C	109.00
C1—Ru—C14	159.60 (7)	H9A—C9—H9B	109.00
C1—Ru—C15	128.85 (7)	H9A—C9—H9C	109.00
C1—Ru—C16	109.48 (6)	H9B—C9—H9C	110.00
C2—Ru—C3	38.48 (6)	C5—C10—H10A	110.00
C2—Ru—C4	64.15 (6)	C5—C10—H10B	109.00
C2—Ru—C5	63.94 (6)	C5—C10—H10C	109.00
C2—Ru—C11	110.39 (6)	H10A—C10—H10B	109.00
C2—Ru—C12	107.49 (7)	H10A—C10—H10C	109.00
C2—Ru—C13	126.18 (7)	H10B—C10—H10C	109.00
C2—Ru—C14	157.01 (7)	Ru—C12—H12	129.00
C2—Ru—C15	166.45 (7)	C11—C12—H12	120.00
C2—Ru—C16	133.93 (6)	C13—C12—H12	120.00
C3—Ru—C4	38.27 (6)	Ru—C13—H13	129.00
C3—Ru—C5	64.10 (6)	C12—C13—H13	120.00
C3—Ru—C11	139.15 (6)	C14—C13—H13	120.00
C3—Ru—C12	113.91 (6)	Ru—C14—H14	130.00
C3—Ru—C13	106.66 (7)	C13—C14—H14	120.00
C3—Ru—C14	121.35 (7)	C15—C14—H14	120.00
C3—Ru—C15	150.37 (6)	Ru—C15—H15	129.00
C3—Ru—C16	172.41 (6)	C14—C15—H15	120.00
C4—Ru—C5	38.34 (6)	C16—C15—H15	120.00
C4—Ru—C11	173.61 (6)	Ru—C16—H16	129.00
C4—Ru—C12	145.85 (6)	C11—C16—H16	119.00
C4—Ru—C13	118.14 (7)	C15—C16—H16	119.00
C4—Ru—C14	107.05 (7)	C11—C17—H17A	109.00
C4—Ru—C15	117.50 (6)	C11—C17—H17B	109.00
C4—Ru—C16	144.44 (6)	C18—C17—H17A	109.00
C5—Ru—C11	137.16 (6)	C18—C17—H17B	109.00
C5—Ru—C12	169.20 (7)	H17A—C17—H17B	109.00
C5—Ru—C13	152.53 (7)	C17—C18—H18A	108.00
C5—Ru—C14	123.52 (7)	C17—C18—H18B	108.00
C5—Ru—C15	108.31 (6)	C19—C18—H18A	108.00
C5—Ru—C16	114.10 (6)	C19—C18—H18B	108.00
C11—Ru—C12	37.27 (6)	H18A—C18—H18B	109.00
C11—Ru—C13	67.49 (7)	C19—C20—H20A	109.00
C11—Ru—C14	79.26 (7)	C19—C20—H20B	109.00
C11—Ru—C15	66.93 (6)	C19—C20—H20C	109.00
C11—Ru—C16	36.96 (6)	H20A—C20—H20B	109.00
C12—Ru—C13	37.68 (8)	H20A—C20—H20C	109.00
C12—Ru—C14	67.00 (8)	H20B—C20—H20C	110.00
C12—Ru—C15	78.68 (7)	C22—C21—C26	115.00 (14)
C12—Ru—C16	66.35 (7)	C22—C21—B	121.96 (13)
C13—Ru—C14	36.73 (8)	C26—C21—B	122.94 (13)
C13—Ru—C15	66.04 (7)	C21—C22—C23	122.81 (15)
C13—Ru—C16	78.29 (7)	C22—C23—C24	120.23 (15)
C14—Ru—C15	36.47 (8)	C23—C24—C25	118.62 (16)
C14—Ru—C16	66.06 (7)	C24—C25—C26	120.69 (16)
C15—Ru—C16	36.82 (7)	C21—C26—C25	122.64 (15)
Ru—C1—C2	70.91 (9)	C28—C27—C32	114.66 (15)
Ru—C1—C5	71.38 (9)	C28—C27—B	122.66 (14)
Ru—C1—C6	125.51 (12)	C32—C27—B	122.59 (14)
C2—C1—C5	108.21 (14)	C27—C28—C29	123.16 (16)
C2—C1—C6	126.13 (15)	C28—C29—C30	120.33 (17)
C5—C1—C6	125.61 (15)	C29—C30—C31	118.85 (18)
Ru—C2—C1	70.84 (9)	C30—C31—C32	120.29 (18)
Ru—C2—C3	70.47 (9)	C27—C32—C31	122.67 (17)
Ru—C2—C7	127.43 (11)	C34—C33—C38	115.08 (15)
C1—C2—C3	107.90 (14)	C34—C33—B	122.80 (14)
C1—C2—C7	125.98 (14)	C38—C33—B	121.89 (14)
C3—C2—C7	125.98 (14)	C33—C34—C35	122.81 (18)
Ru—C3—C2	71.05 (9)	C34—C35—C36	120.29 (19)
Ru—C3—C4	71.35 (9)	C35—C36—C37	119.23 (19)
Ru—C3—C8	124.49 (12)	C36—C37—C38	120.4 (2)
C2—C3—C4	108.13 (14)	C33—C38—C37	122.14 (17)
C2—C3—C8	125.40 (15)	C40—C39—C44	114.74 (14)
C4—C3—C8	126.46 (15)	C40—C39—B	123.72 (14)
Ru—C4—C3	70.37 (9)	C44—C39—B	121.30 (14)
Ru—C4—C5	71.07 (9)	C39—C40—C41	122.51 (17)
Ru—C4—C9	126.69 (12)	C40—C41—C42	120.66 (18)
C3—C4—C5	107.81 (14)	C41—C42—C43	118.81 (17)
C3—C4—C9	126.81 (14)	C42—C43—C44	120.17 (17)
C5—C4—C9	125.29 (14)	C39—C44—C43	123.10 (15)
Ru—C5—C1	70.44 (9)	C23—C22—H22	119.00
Ru—C5—C4	70.59 (9)	C21—C22—H22	119.00
Ru—C5—C10	128.02 (12)	C22—C23—H23	120.00
C1—C5—C4	107.94 (14)	C24—C23—H23	120.00
C1—C5—C10	126.20 (15)	C25—C24—H24	121.00
C4—C5—C10	125.71 (14)	C23—C24—H24	121.00
Ru—C11—C12	70.69 (10)	C24—C25—H25	120.00
Ru—C11—C16	70.86 (9)	C26—C25—H25	120.00
Ru—C11—C17	129.51 (12)	C21—C26—H26	119.00
C12—C11—C16	117.83 (15)	C25—C26—H26	119.00
C12—C11—C17	121.77 (16)	C29—C28—H28	118.00
C16—C11—C17	120.41 (15)	C27—C28—H28	118.00
Ru—C12—C11	72.05 (10)	C28—C29—H29	120.00
Ru—C12—C13	70.95 (12)	C30—C29—H29	120.00
C11—C12—C13	119.99 (17)	C29—C30—H30	121.00
Ru—C13—C12	71.38 (11)	C31—C30—H30	121.00
Ru—C13—C14	72.06 (12)	C32—C31—H31	120.00
C12—C13—C14	120.16 (18)	C30—C31—H31	120.00
Ru—C14—C13	71.21 (11)	C27—C32—H32	119.00
Ru—C14—C15	71.70 (10)	C31—C32—H32	119.00
C13—C14—C15	120.11 (18)	C33—C34—H34	119.00
Ru—C15—C14	71.83 (11)	C35—C34—H34	119.00
Ru—C15—C16	71.48 (10)	C36—C35—H35	120.00
C14—C15—C16	120.20 (18)	C34—C35—H35	120.00
Ru—C16—C11	72.19 (9)	C35—C36—H36	120.00
Ru—C16—C15	71.70 (11)	C37—C36—H36	120.00
C11—C16—C15	121.72 (16)	C38—C37—H37	120.00
C11—C17—C18	111.78 (14)	C36—C37—H37	120.00
C17—C18—C19	114.54 (15)	C33—C38—H38	119.00
O19—C19—C18	122.43 (17)	C37—C38—H38	119.00
O19—C19—C20	121.50 (19)	C39—C40—H40	119.00
C18—C19—C20	116.05 (18)	C41—C40—H40	119.00
C1—C6—H6A	109.00	C42—C41—H41	120.00
C1—C6—H6B	109.00	C40—C41—H41	120.00
C1—C6—H6C	110.00	C41—C42—H42	121.00
H6A—C6—H6B	109.00	C43—C42—H42	121.00
H6A—C6—H6C	109.00	C42—C43—H43	120.00
H6B—C6—H6C	109.00	C44—C43—H43	120.00
C2—C7—H7A	109.00	C43—C44—H44	118.00
C2—C7—H7B	109.00	C39—C44—H44	118.00
C2—C7—H7C	109.00	C21—B—C33	108.06 (12)
H7A—C7—H7B	109.00	C21—B—C39	112.13 (13)
H7A—C7—H7C	110.00	C27—B—C39	108.69 (12)
H7B—C7—H7C	109.00	C33—B—C39	107.53 (12)
C3—C8—H8A	109.00	C27—B—C33	111.59 (13)
C3—C8—H8B	109.00	C21—B—C27	108.87 (12)
			
C2—Ru—C1—C5	117.74 (13)	C1—Ru—C15—C16	−68.82 (13)
C2—Ru—C1—C6	−121.30 (18)	C3—Ru—C15—C14	52.49 (19)
C3—Ru—C1—C2	−37.61 (9)	C3—Ru—C15—C16	−175.58 (12)
C3—Ru—C1—C5	80.13 (10)	C4—Ru—C15—C14	81.46 (13)
C3—Ru—C1—C6	−158.91 (16)	C4—Ru—C15—C16	−146.61 (10)
C4—Ru—C1—C2	−80.31 (10)	C5—Ru—C15—C14	122.07 (12)
C4—Ru—C1—C5	37.42 (9)	C5—Ru—C15—C16	−106.00 (11)
C4—Ru—C1—C6	158.38 (16)	C11—Ru—C15—C14	−103.62 (13)
C5—Ru—C1—C2	−117.74 (13)	C11—Ru—C15—C16	28.31 (10)
C5—Ru—C1—C6	120.96 (18)	C12—Ru—C15—C14	−66.46 (13)
C11—Ru—C1—C2	98.44 (10)	C12—Ru—C15—C16	65.48 (11)
C11—Ru—C1—C5	−143.82 (9)	C13—Ru—C15—C14	−29.05 (13)
C11—Ru—C1—C6	−22.86 (16)	C13—Ru—C15—C16	102.88 (12)
C12—Ru—C1—C2	62.58 (12)	C14—Ru—C15—C16	131.93 (17)
C12—Ru—C1—C5	−179.68 (9)	C16—Ru—C15—C14	−131.93 (17)
C12—Ru—C1—C6	−58.72 (17)	C1—Ru—C16—C11	−96.92 (10)
C14—Ru—C1—C2	−148.7 (2)	C1—Ru—C16—C15	129.62 (11)
C14—Ru—C1—C5	−31.0 (2)	C2—Ru—C16—C11	−61.60 (12)
C14—Ru—C1—C6	90.0 (3)	C2—Ru—C16—C15	164.94 (10)
C15—Ru—C1—C2	174.09 (9)	C4—Ru—C16—C11	−169.45 (10)
C15—Ru—C1—C5	−68.17 (12)	C4—Ru—C16—C15	57.08 (15)
C15—Ru—C1—C6	52.79 (17)	C5—Ru—C16—C11	−137.87 (9)
C16—Ru—C1—C2	137.74 (9)	C5—Ru—C16—C15	88.67 (11)
C16—Ru—C1—C5	−104.53 (10)	C11—Ru—C16—C15	−133.47 (15)
C16—Ru—C1—C6	16.43 (16)	C12—Ru—C16—C11	30.33 (10)
C1—Ru—C2—C3	−117.94 (13)	C12—Ru—C16—C15	−103.13 (12)
C1—Ru—C2—C7	121.16 (18)	C13—Ru—C16—C11	68.00 (10)
C3—Ru—C2—C1	117.94 (13)	C13—Ru—C16—C15	−65.47 (12)
C3—Ru—C2—C7	−120.90 (18)	C14—Ru—C16—C11	104.53 (11)
C4—Ru—C2—C1	80.35 (10)	C14—Ru—C16—C15	−28.93 (11)
C4—Ru—C2—C3	−37.59 (9)	C15—Ru—C16—C11	133.47 (15)
C4—Ru—C2—C7	−158.49 (16)	C15—Ru—C14—H14	114.00
C5—Ru—C2—C1	37.51 (9)	C16—Ru—C14—H14	143.00
C5—Ru—C2—C3	−80.43 (10)	C1—Ru—C15—H15	45.00
C5—Ru—C2—C7	158.67 (16)	C3—Ru—C15—H15	−62.00
C11—Ru—C2—C1	−96.04 (10)	C4—Ru—C15—H15	−33.00
C11—Ru—C2—C3	146.02 (9)	C1—Ru—C12—H12	−49.00
C11—Ru—C2—C7	25.12 (16)	C2—Ru—C12—H12	−14.00
C12—Ru—C2—C1	−135.41 (10)	C3—Ru—C12—H12	27.00
C12—Ru—C2—C3	106.65 (10)	C4—Ru—C12—H12	56.00
C12—Ru—C2—C7	−14.25 (16)	C11—Ru—C12—H12	−115.00
C13—Ru—C2—C1	−172.50 (10)	C13—Ru—C12—H12	114.00
C13—Ru—C2—C3	69.56 (12)	C14—Ru—C12—H12	143.00
C13—Ru—C2—C7	−51.34 (17)	C15—Ru—C12—H12	179.00
C14—Ru—C2—C1	152.42 (17)	C16—Ru—C12—H12	−145.00
C14—Ru—C2—C3	34.5 (2)	C2—Ru—C13—H13	−44.00
C14—Ru—C2—C7	−86.4 (2)	C3—Ru—C13—H13	−6.00
C16—Ru—C2—C1	−61.69 (12)	C4—Ru—C13—H13	34.00
C16—Ru—C2—C3	−179.63 (9)	C5—Ru—C13—H13	59.00
C16—Ru—C2—C7	59.47 (17)	C11—Ru—C13—H13	−143.00
C1—Ru—C3—C2	37.38 (9)	C12—Ru—C13—H13	−114.00
C1—Ru—C3—C4	−80.21 (10)	C14—Ru—C13—H13	114.00
C1—Ru—C3—C8	157.85 (16)	C15—Ru—C13—H13	143.00
C2—Ru—C3—C4	−117.59 (13)	C16—Ru—C13—H13	180.00
C2—Ru—C3—C8	120.47 (18)	C1—Ru—C14—H14	62.00
C4—Ru—C3—C2	117.59 (13)	C2—Ru—C14—H14	−63.00
C4—Ru—C3—C8	−121.94 (18)	C3—Ru—C14—H14	−39.00
C5—Ru—C3—C2	79.98 (10)	C4—Ru—C14—H14	1.00
C5—Ru—C3—C4	−37.61 (9)	C5—Ru—C14—H14	39.00
C5—Ru—C3—C8	−159.55 (16)	C11—Ru—C14—H14	180.00
C11—Ru—C3—C2	−53.22 (13)	C12—Ru—C14—H14	−144.00
C11—Ru—C3—C4	−170.81 (9)	C13—Ru—C14—H14	−114.00
C11—Ru—C3—C8	67.25 (17)	C11—Ru—C16—H16	113.00
C12—Ru—C3—C2	−88.31 (10)	C12—Ru—C16—H16	144.00
C12—Ru—C3—C4	154.10 (9)	C13—Ru—C16—H16	−179.00
C12—Ru—C3—C8	32.16 (16)	C14—Ru—C16—H16	−142.00
C13—Ru—C3—C2	−127.86 (10)	C15—Ru—C16—H16	−113.00
C13—Ru—C3—C4	114.55 (10)	C5—Ru—C15—H15	8.00
C13—Ru—C3—C8	−7.39 (16)	C11—Ru—C15—H15	142.00
C14—Ru—C3—C2	−164.99 (10)	C12—Ru—C15—H15	179.00
C14—Ru—C3—C4	77.42 (11)	C13—Ru—C15—H15	−143.00
C14—Ru—C3—C8	−44.52 (17)	C14—Ru—C15—H15	−114.00
C15—Ru—C3—C2	161.50 (12)	C16—Ru—C15—H15	114.00
C15—Ru—C3—C4	43.91 (17)	C1—Ru—C16—H16	17.00
C15—Ru—C3—C8	−78.03 (19)	C2—Ru—C16—H16	52.00
C1—Ru—C4—C3	80.49 (10)	C4—Ru—C16—H16	−56.00
C1—Ru—C4—C5	−37.26 (9)	C5—Ru—C16—H16	−24.00
C1—Ru—C4—C9	−157.67 (16)	Ru—C1—C2—C3	61.04 (11)
C2—Ru—C4—C3	37.80 (9)	Ru—C1—C2—C7	−122.88 (16)
C2—Ru—C4—C5	−79.96 (10)	C5—C1—C2—Ru	−62.00 (11)
C2—Ru—C4—C9	159.64 (16)	C5—C1—C2—C3	−0.97 (18)
C3—Ru—C4—C5	−117.75 (13)	C5—C1—C2—C7	175.12 (15)
C3—Ru—C4—C9	121.85 (18)	C2—C1—C5—Ru	61.70 (11)
C5—Ru—C4—C3	117.75 (13)	C2—C1—C5—C4	0.76 (18)
C5—Ru—C4—C9	−120.40 (18)	C6—C1—C2—Ru	120.56 (17)
C12—Ru—C4—C3	−45.35 (16)	C6—C1—C2—C3	−178.40 (15)
C12—Ru—C4—C5	−163.10 (12)	C6—C1—C2—C7	−2.3 (3)
C12—Ru—C4—C9	76.50 (18)	Ru—C1—C5—C4	−60.95 (11)
C13—Ru—C4—C3	−81.20 (11)	Ru—C1—C5—C10	123.37 (16)
C13—Ru—C4—C5	161.05 (10)	C6—C1—C5—Ru	−120.84 (16)
C13—Ru—C4—C9	40.65 (16)	C6—C1—C5—C4	178.21 (15)
C14—Ru—C4—C3	−119.33 (10)	C2—C1—C5—C10	−174.93 (15)
C14—Ru—C4—C5	122.92 (10)	C6—C1—C5—C10	2.5 (3)
C14—Ru—C4—C9	2.52 (16)	C1—C2—C3—C4	0.81 (18)
C15—Ru—C4—C3	−157.26 (9)	C1—C2—C3—C8	179.35 (16)
C15—Ru—C4—C5	84.99 (11)	Ru—C2—C3—C4	62.08 (11)
C15—Ru—C4—C9	−35.42 (16)	Ru—C2—C3—C8	−119.38 (17)
C16—Ru—C4—C3	168.18 (10)	C1—C2—C3—Ru	−61.28 (11)
C16—Ru—C4—C5	50.43 (14)	C7—C2—C3—C8	3.3 (3)
C16—Ru—C4—C9	−69.97 (18)	C7—C2—C3—Ru	122.64 (16)
C1—Ru—C5—C4	118.14 (13)	C7—C2—C3—C4	−175.28 (15)
C1—Ru—C5—C10	−121.18 (18)	C2—C3—C4—C5	−0.34 (18)
C2—Ru—C5—C1	−37.58 (9)	Ru—C3—C4—C5	61.55 (11)
C2—Ru—C5—C4	80.56 (10)	Ru—C3—C4—C9	−121.70 (17)
C2—Ru—C5—C10	−158.77 (16)	C2—C3—C4—Ru	−61.89 (11)
C3—Ru—C5—C1	−80.60 (10)	C8—C3—C4—C5	−178.86 (16)
C3—Ru—C5—C4	37.55 (9)	C2—C3—C4—C9	176.41 (16)
C3—Ru—C5—C10	158.22 (16)	C8—C3—C4—Ru	119.59 (17)
C4—Ru—C5—C1	−118.14 (13)	C8—C3—C4—C9	−2.1 (3)
C4—Ru—C5—C10	120.67 (18)	C3—C4—C5—C1	−0.26 (18)
C11—Ru—C5—C1	54.89 (12)	Ru—C4—C5—C1	60.85 (11)
C11—Ru—C5—C4	173.03 (9)	Ru—C4—C5—C10	−123.44 (16)
C11—Ru—C5—C10	−66.30 (17)	C3—C4—C5—Ru	−61.11 (11)
C13—Ru—C5—C1	−156.52 (14)	C9—C4—C5—Ru	122.08 (16)
C13—Ru—C5—C4	−38.37 (18)	C9—C4—C5—C1	−177.07 (15)
C13—Ru—C5—C10	82.3 (2)	C3—C4—C5—C10	175.45 (15)
C14—Ru—C5—C1	167.57 (10)	C9—C4—C5—C10	−1.4 (3)
C14—Ru—C5—C4	−74.29 (12)	C12—C11—C17—C18	−78.6 (2)
C14—Ru—C5—C10	46.38 (17)	C16—C11—C17—C18	101.5 (2)
C15—Ru—C5—C1	130.40 (10)	Ru—C11—C12—C13	−54.33 (17)
C15—Ru—C5—C4	−111.46 (10)	C16—C11—C12—Ru	54.70 (14)
C15—Ru—C5—C10	9.22 (16)	C16—C11—C12—C13	0.4 (3)
C16—Ru—C5—C1	91.27 (10)	C17—C11—C12—Ru	−125.17 (16)
C16—Ru—C5—C4	−150.59 (9)	C17—C11—C12—C13	−179.50 (17)
C16—Ru—C5—C10	−29.92 (16)	Ru—C11—C16—C15	54.11 (15)
C1—Ru—C11—C12	−133.13 (11)	C12—C11—C16—Ru	−54.62 (14)
C1—Ru—C11—C16	96.69 (10)	C12—C11—C16—C15	−0.5 (3)
C1—Ru—C11—C17	−17.40 (17)	C17—C11—C16—Ru	125.26 (15)
C2—Ru—C11—C12	−92.34 (11)	C17—C11—C16—C15	179.36 (16)
C2—Ru—C11—C16	137.48 (9)	Ru—C11—C17—C18	−168.97 (12)
C2—Ru—C11—C17	23.39 (17)	Ru—C12—C13—C14	−55.12 (18)
C3—Ru—C11—C12	−60.21 (14)	C11—C12—C13—Ru	54.85 (16)
C3—Ru—C11—C16	169.61 (9)	C11—C12—C13—C14	−0.3 (3)
C3—Ru—C11—C17	55.51 (19)	Ru—C13—C14—C15	−54.51 (17)
C5—Ru—C11—C12	−165.58 (11)	C12—C13—C14—Ru	54.80 (18)
C5—Ru—C11—C16	64.24 (12)	C12—C13—C14—C15	0.3 (3)
C5—Ru—C11—C17	−49.85 (19)	Ru—C14—C15—C16	−54.70 (16)
C12—Ru—C11—C16	−130.18 (15)	C13—C14—C15—Ru	54.28 (17)
C12—Ru—C11—C17	115.7 (2)	C13—C14—C15—C16	−0.4 (3)
C13—Ru—C11—C12	29.51 (11)	Ru—C15—C16—C11	−54.33 (15)
C13—Ru—C11—C16	−100.68 (11)	C14—C15—C16—Ru	54.87 (17)
C13—Ru—C11—C17	145.23 (18)	C14—C15—C16—C11	0.5 (3)
C14—Ru—C11—C12	65.96 (12)	C11—C17—C18—C19	−174.24 (16)
C14—Ru—C11—C16	−64.23 (11)	C17—C18—C19—O19	11.2 (3)
C14—Ru—C11—C17	−178.32 (17)	C17—C18—C19—C20	−170.24 (19)
C15—Ru—C11—C12	101.96 (12)	C26—C21—C22—C23	−0.7 (2)
C15—Ru—C11—C16	−28.22 (10)	B—C21—C22—C23	175.79 (15)
C15—Ru—C11—C17	−142.31 (18)	C22—C21—C26—C25	0.6 (2)
C16—Ru—C11—C12	130.18 (15)	B—C21—C26—C25	−175.91 (15)
C16—Ru—C11—C17	−114.09 (19)	C22—C21—B—C27	−97.48 (16)
C1—Ru—C12—C11	65.73 (13)	C22—C21—B—C33	23.88 (19)
C1—Ru—C12—C13	−162.38 (10)	C22—C21—B—C39	142.22 (14)
C2—Ru—C12—C11	100.91 (10)	C26—C21—B—C27	78.77 (17)
C2—Ru—C12—C13	−127.20 (11)	C26—C21—B—C33	−159.87 (14)
C3—Ru—C12—C11	141.62 (10)	C26—C21—B—C39	−41.54 (19)
C3—Ru—C12—C13	−86.49 (12)	C21—C22—C23—C24	0.5 (2)
C4—Ru—C12—C11	170.43 (10)	C22—C23—C24—C25	0.0 (2)
C4—Ru—C12—C13	−57.68 (17)	C23—C24—C25—C26	−0.2 (2)
C11—Ru—C12—C13	131.89 (16)	C24—C25—C26—C21	−0.1 (2)
C13—Ru—C12—C11	−131.89 (16)	C32—C27—C28—C29	1.1 (2)
C14—Ru—C12—C11	−102.92 (12)	B—C27—C28—C29	−175.49 (14)
C14—Ru—C12—C13	28.97 (12)	C28—C27—C32—C31	−2.3 (2)
C15—Ru—C12—C11	−66.62 (11)	B—C27—C32—C31	174.28 (16)
C15—Ru—C12—C13	65.27 (11)	C28—C27—B—C21	−24.56 (19)
C16—Ru—C12—C11	−30.09 (9)	C28—C27—B—C33	−143.74 (14)
C16—Ru—C12—C13	101.80 (12)	C28—C27—B—C39	97.85 (17)
C2—Ru—C13—C12	70.25 (13)	C32—C27—B—C21	159.11 (14)
C2—Ru—C13—C14	−157.95 (12)	C32—C27—B—C33	39.9 (2)
C3—Ru—C13—C12	107.74 (11)	C32—C27—B—C39	−78.48 (18)
C3—Ru—C13—C14	−120.46 (13)	C27—C28—C29—C30	0.8 (3)
C4—Ru—C13—C12	147.46 (10)	C28—C29—C30—C31	−1.6 (3)
C4—Ru—C13—C14	−80.75 (14)	C29—C30—C31—C32	0.5 (3)
C5—Ru—C13—C12	173.35 (12)	C30—C31—C32—C27	1.6 (3)
C5—Ru—C13—C14	−54.9 (2)	C38—C33—C34—C35	0.2 (3)
C11—Ru—C13—C12	−29.20 (10)	B—C33—C34—C35	174.77 (17)
C11—Ru—C13—C14	102.59 (13)	C34—C33—C38—C37	−0.6 (3)
C12—Ru—C13—C14	131.79 (18)	B—C33—C38—C37	−175.18 (17)
C14—Ru—C13—C12	−131.79 (18)	C34—C33—B—C21	−89.42 (18)
C15—Ru—C13—C12	−102.95 (12)	C34—C33—B—C27	30.2 (2)
C15—Ru—C13—C14	28.85 (12)	C34—C33—B—C39	149.34 (15)
C16—Ru—C13—C12	−66.31 (11)	C38—C33—B—C21	84.77 (18)
C16—Ru—C13—C14	65.48 (13)	C38—C33—B—C27	−155.57 (15)
C1—Ru—C14—C13	175.54 (17)	C38—C33—B—C39	−36.5 (2)
C1—Ru—C14—C15	−52.4 (3)	C33—C34—C35—C36	0.2 (3)
C2—Ru—C14—C13	50.9 (2)	C34—C35—C36—C37	−0.3 (3)
C2—Ru—C14—C15	−177.03 (15)	C35—C36—C37—C38	−0.1 (3)
C3—Ru—C14—C13	75.22 (14)	C36—C37—C38—C33	0.5 (3)
C3—Ru—C14—C15	−152.67 (11)	C44—C39—C40—C41	0.8 (2)
C4—Ru—C14—C13	114.44 (13)	B—C39—C40—C41	−173.63 (16)
C4—Ru—C14—C15	−113.44 (12)	C40—C39—C44—C43	−1.3 (2)
C5—Ru—C14—C13	153.10 (11)	B—C39—C44—C43	173.25 (15)
C5—Ru—C14—C15	−74.79 (14)	C40—C39—B—C21	−28.5 (2)
C11—Ru—C14—C13	−66.59 (13)	C40—C39—B—C27	−148.89 (15)
C11—Ru—C14—C15	65.52 (12)	C40—C39—B—C33	90.16 (18)
C12—Ru—C14—C13	−29.67 (12)	C44—C39—B—C21	157.45 (13)
C12—Ru—C14—C15	102.44 (13)	C44—C39—B—C27	37.04 (19)
C13—Ru—C14—C15	132.11 (19)	C44—C39—B—C33	−83.91 (17)
C15—Ru—C14—C13	−132.11 (19)	C39—C40—C41—C42	0.1 (3)
C16—Ru—C14—C13	−102.91 (14)	C40—C41—C42—C43	−0.5 (3)
C16—Ru—C14—C15	29.20 (11)	C41—C42—C43—C44	0.0 (3)
C1—Ru—C15—C14	159.25 (12)	C42—C43—C44—C39	1.0 (3)

Symmetry codes: (i) −*x*+1, −*y*, −*z*; (ii) *x*+1/2, −*y*+1/2, *z*−1/2; (iii) *x*+1, *y*, *z*; (iv) *x*+1/2, −*y*+1/2, *z*+1/2; (v) −*x*+1/2, *y*−1/2, −*z*+1/2; (vi) *x*−1, *y*, *z*; (vii) *x*−1/2, −*y*+1/2, *z*−1/2; (viii) −*x*+1/2, *y*+1/2, −*z*+1/2; (ix) *x*−1/2, −*y*+1/2, *z*+1/2; (x) −*x*, −*y*, −*z*; (xi) −*x*, −*y*+1, −*z*.
